# Effect of moisture content variation on dielectric properties of seeds of multipurpose species in 20 MHz-3 GHz frequency range

**DOI:** 10.1038/s41598-025-32111-w

**Published:** 2025-12-24

**Authors:** Małgorzata Budzeń, Marcin Kafarski, Jacek Majcher, Agnieszka Szypłowska, Andrzej Wilczek, Arkadiusz Lewandowski, Tomasz Leśniak, Lech Gałęzewski

**Affiliations:** 1 Institute of Agrophysics, Polish Academy of Sciences, Doświadczalna 4, Lublin, 20-290 Poland; 2 Department of Electrical Engineering and Smart Technologies, Lublin University of Technology, Nadbystrzycka 38A, Lublin, 20-618 Poland; 3 Institute of Electronic Systems, Warsaw University of Technology, Nowowiejska 15/19, Warsaw, 00-665 Poland; 4 E-test Sp. z o.o, Stasin 90, Motycz, 21-030 Poland; 5 Department of Agronomy, Faculty of Agriculture and Biotechnology, Bydgoszcz University of Science and Technology, 7 Prof. S. Kaliskiego St, Bydgoszcz, 85-796 Poland

**Keywords:** Seeds of multipurpose crop species, Volumetric water content, Dielectric permittivity, Loss tangent, Penetration depth, Electrical conductivity, Engineering, Materials science, Physics, Plant sciences

## Abstract

The study presents measurements and analysis of complex dielectric permittivity spectra of seeds of multipurpose crop species such as mustard, buckwheat, phacelia, and crimson clover. These species due to their ecosystem service properties such as the improvement of soil health, carbon sequestration and pollinator support, can be part of sustainable food production.Seeds with different moisture content were measured in a coaxial cell system covering 20 MHz to 3 GHz frequency range. The results indicate that dielectric parameters such as the square root of dielectric permittivity, loss tangent, penetration depth, and effective electrical conductivity are influenced by factors including volumetric water content and measurement frequency. Additionally, significant variations in dielectric properties are observed among different seed types and species. Dielectric permittivity, in particular, shows potential as a reliable indicator of seed water content, making it a valuable tool for assessing seed quality and monitoring changes during storage under various conditions. These findings highlight the importance of dielectric measurements in seed quality assessment and storage management as well provide valuable information for modelling and designing efficient dielectric heating and microwave-assisted conditions.

## Introduction

Modern food systems are increasingly threatened by biodiversity loss, soil degradation, and the impact of climate change. This necessitates a shift toward sustainable, multifunctional cropping systems that integrate food production with ecosystem services such as improving soil health, carbon sequestration, and pollinator suport^1,2^.

Seeds play a crucial role in the evolution and survival of higher plants as they are basic propagation units^3^. The most important factor affecting seed harvest, storage, processing and seed trade is the moisture content^4^. The amount of moisture in seeds affects many processes and can be summarized as follows: seed moisture above 45–60% - germination occurs, 18–20% - heating may occur, 12–14% molds grow on and in seed, below 8–9% - little or no insect activity, 4–8% - sealed storage is safe^5^. Critical moisture values vary from species to species, as individual species have their own moisture-absorbing and moisture-holding capability^6^. The moisture content of seeds during storage is the most influential factor affecting their longevity^7^. After harvest, particular attention should be paid to reduce seed moisture content to a safe level for storage^8^. On the other hand, too low moisture content can cause mechanical breakage and related injuries. This can lead to fungal attack, reduced storage potential and an irreversible loss of seed technological parameters^6^.

Accurate knowledge of the amount of water contained in the seeds is essential to estimate their commercial value. Therefore it is of great significance to select an appropriate, non-destructive seed moisture content testing method. The Frequency Domain Reflectometry (FDR)^9–11^ and the Frequency Domain Transmissiometry (FDT)^12^ are among the safe and nondestructive dielectric spectroscopy methods that can be applied to investigate the dielectric properties and their dependence on moisture for various agricultural materials. The use of the coaxial transmission line method is a promising approach to transmission measurements aimed at determining the complex dielectric permittivity spectrum (ε*) of heterogeneous agricultural materials such as soil or seeds^13–17^. It is a well-known fact that dielectric permittivity depends on water content of the material. The linear relationship between water content and the square root of the real part of the dielectric permittivity of soil is well established in soil physics and electromagnetic studies^18^. Dielectric properties are crucial to understanding how electromagnetic field interacts with materials. As reported by^19,20^ the dielectric properties may reflect physical and structural changes following the application of various processing methods to agri-food materials. Changes in the structure and chemical composition of the material affect the movement and behaviour of polar water molecules or ionic conductivity. This can be reflected in the dielectric properties of materials, which are described by frequency-dependent complex relative dielectric permittivity: ε* = *ε*′ *− jε*″. Complex permittivity includes two components: the real part *ε*′ and the imaginary part *ε*″, where *j* is the imaginary unit^21^. The real part *ε*′ and imaginary part *ε*″ of complex dielectric permittivity reflect the ability of a material for storing and dissipating the energy of the electric field, respectively^22^. Gupta et al.^23^ found that real and imaginary part of dielectric permittivity of gluten-free grains increase with the increase in moisture content. Chauhan et al.^24^ had similar observations for corn, jowar, ashoka and banana leaves. Authors^23^ also concluded that for higher moisture there are more free molecules in comparison to bound water molecules. Loss tangent is the ratio between the imaginary part of permittivity and real part of permittivity. Loss tangent can be used to describe the ability of the material to convert the energy of the electric field into heat at a specific frequency and temperature^19,21,25–27^. From literature reports by^26,28^ materials with moderate value of ε′, and higher value of ε″ (and so high values of tanδ), would convert more energy into heat than materials with a lower value of ε″. Therefore the knowledge of dielectric properties of materials is needed for the application of radio-frequency/microwave heating^29–32^. Determination of the conductivity properties (the electrical conductivity test) of the seeds is a quick and practical method of assessing seed vigour. The method is related to the assessment of the loss of the cell membrane integrity system, which is one of the first changes that occurs as a result of seed deterioration^33,34^. The higher the rate of recovery of the integrity of the cell membrane system during seed imbibition, the lower will be the leaching of electrolytes into the external environment and thus the electrical conductivity, indicating higher vigour^35^.

The study of the dielectric properties and their interrelationships plays a significant role in observing mass and heat exchange. This is important not only for the non-destructive determination of the moisture content in seeds but also for seed drying processess or the assessment of seeds viability, and is of both practical and theoretical importance.The agricultural usefulness of the plant species considered below motivates further need and importance of in-depth seed dielectric testing. The results can be helpful in planning post-harvest radio-frequency and microwave drying and processing as well as in optimization of storage conditions and quality control of the seeds. Microwave radiation is one of the factors improving the seed germination process^36–38^. Therefore, knowledge of the dielectric properties of these plant seeds can also be used as a basis for designing microwave refinement/stimulation processes of the seeds. Seeds from the following plants were selected for the study: mustard, buckwheat, phacelia and crimson clover.

Mustard is a plant eagerly cultivated by farmers, due to its good adaptability across various agroclimatic conditions and its high yield. The mustard seeds are usually about 1 to 2 millimetres in diameter, composed mostly of oil (28–42%), followed by protein (25–40%), carbohydrate (15–35%), fiber (10–15%) and minerals (5–10%)^39^. Mustard seeds are a valuable source of many bioactive components such as polyunsaturated fatty acids and antioxidants such as carotenoids, tocopherols and phenolic compounds. This makes them very popular in food production and other industries^39,40^. White mustard (*Sinapis alba* L.) seed oil can be used for cooking, food preservation, body and hair revitalization, biodiesel production, and as a diesel fuel additive and alternative biofuel^41^. The white mustard is also good cover crop. Gajewski et al.^42^ stated beneficial effects of these cover crops and conventional plough tillage on the majority of the soil properties. There are also reports of conversion of mustard seed residues such as husk during microwave pyrolysis into an energy-rich product^27^. According to Grygier^43^ mustard seeds can also be a source of a natural coagulant that can potentially be used for water treatment, especially in areas where drinking water is scarce.

Buckwheat is a pseudo-cereal processed into products such as breakfast foods, flour and noodles^44^. This plant can be used as livestock forage and feed^45^, green manure, cover crop and melliferous plant^46^. Buckwheat waste, in turn, can be used to produce goods in the fertiliser, food, chemical, pharmaceutical, fabric, yarn and thread, cement, and other sectors, thus contributing to the circular economy^47^. For example buckwheat husk can be used as filling in therapeutic pillows, mattresses and seats^48^. The buckwheat seed is a fruit in the form of an achene, triangular-shaped and dark brown with a shiny texture^49^. They vary in size from about 4 mm at the maximum width and 6 mm long to 2 mm wide and 4 mm long^50^. Buckwheat/whole groats contain mainly starch (55%), protein (12%), lipid (4%), soluble carbohydrates (2%), total dietary fiber (7%) and ash (2%). Other components such as organic acids, phenolic compounds, tannins, phosphorylated sugars, nucleotides and nucleic acids account for 18%^51^. Nowadays, interest in buckwheat-based food increased due to their beneficial properties for human health, gluten-free composition and nutritional value (rich content of proteins, vitamins, minerals, dietary fibers, and bioactive compounds)^49,52–54^. Moreover as reported by Singh et al.^55^ buckwheat (Fagopyrum sp.) is one of the crops containing diverse genetic resources for future agriculture due to their suitability to marginalized environments.

*Phacelia tanacetifolia* Benth. is among the world’s leading honey plants^2,56^, and a good phytosanitary forage plant^57,58^. Phacelia seeds are small, typically around 2.7 × 1.5 mm in size^59^, and dark brown with a coarse, wrinkled, and pitted texture. The main advantage of this plant is its high resistance to drought and frost, phacelia is also one of the best cover crops for improving soil quality^2^. From literature reports it is known that cover crops such as phacelia and buckwheat affect soil carbon levels and enhance the stability of wet aggregates. This confirms the substantial influence of these cover crop residues on soil physical properties^60^. The study by Fernando et al.^61^ demonstrated benefits in soil moisture and soil health aspects with the use of a native species, phacelia tanacetifolia in a semi-arid, Mediterranean climate, specifically the Central Valley of California. Crop biodiversity is the basis for resilient food systems.

The latest reports^2^ indicate that efforts are being made to introduce phacelia on a larger scale in order to eliminate monoculture and significantly increase biodiversity in a given area.

Crimson clover is a good source of nectar for honey production by bees, a green manure and a forage crop^62^ and can also be used in lawns^63^. Moreover, crimson clover (*Trifolium incarnatum* L.) is gaining popularity as cover crop mixtures^64–66^. Crimson clover seeds (*Trifolium incarnatum*) are ~ 2.5 × 1.5 mm, cream to light brown, and oval to spherical in shape^67^.

All these plants can be used for cover crops, which is one of the climate-friendly agricultural practices used to increase soil organic carbon sequestration. This makes them a valuable option for sustainable agriculture.They are not only a source of food, but also contributes to the development of various industries. The species diversity is an added advantage of the research in the context of increasing the abundance of pollinating insects in agricultural landscapes. Literature report indicate that the all presented plant species play significant role in agriculture due to its versatile uses and economic potential. Therefore, monitoring and controlling moisture content using non-destructive dielectric spectroscopy method for these seed species is essential to maximize seed longevity, quality, and market value. The proper moisture content for safest harvesting varies from crop to crop^5^. The moisture content of buckwheat grains after harvest usually reaches 20–25%. This necessitates immediate drying of the grain mass, which prevents it from overheating and becoming musty. For longer-term storage, buckwheat grain should be at no more than 12 to 13% moisture content^50^. Phacelia harvested seed material must be dried to prevent mold and decay. The recommended target moisture content of phacelia seeds is 12%^68^, and for air-free moisture conditions for long-term storage of mustard seeds and crimson clover is 7% and 8% respectively, according to^69^.

Data on dielectric properties of these seeds under different moisture conditions are lacking in literature. In view of the above the aim of the study is to investigate dielectric properties in the frequency range of 20 MHz-3 GHz: complex dielectric permittivity, loss tangent, penetration depth and also electrical conductivity of the seeds of multipurpose crop species such as mustard, buckwheat, phacelia, and crimson clover under moisture content variation that can occur between harvest and storage. The data obtained in this study can be used to assess seed quality, assist in storage management, and provide valuable information for potential modelling and design of efficient dielectric and microwave-assisted heating conditions.

## Materials and methods

### Seed material

The following plants seed material collected in 2024 was examined:

- Mustard (*Sinapis alba* L.)
- Common buckwheat (*Fagopyrum esculentum Moench* L.)
- Tansy phacelia (*Phacelia tanacetifolia Benth*. L.)
- Crimson clover (*Trifolium incarnatum* L.)

**Fig. 1:**
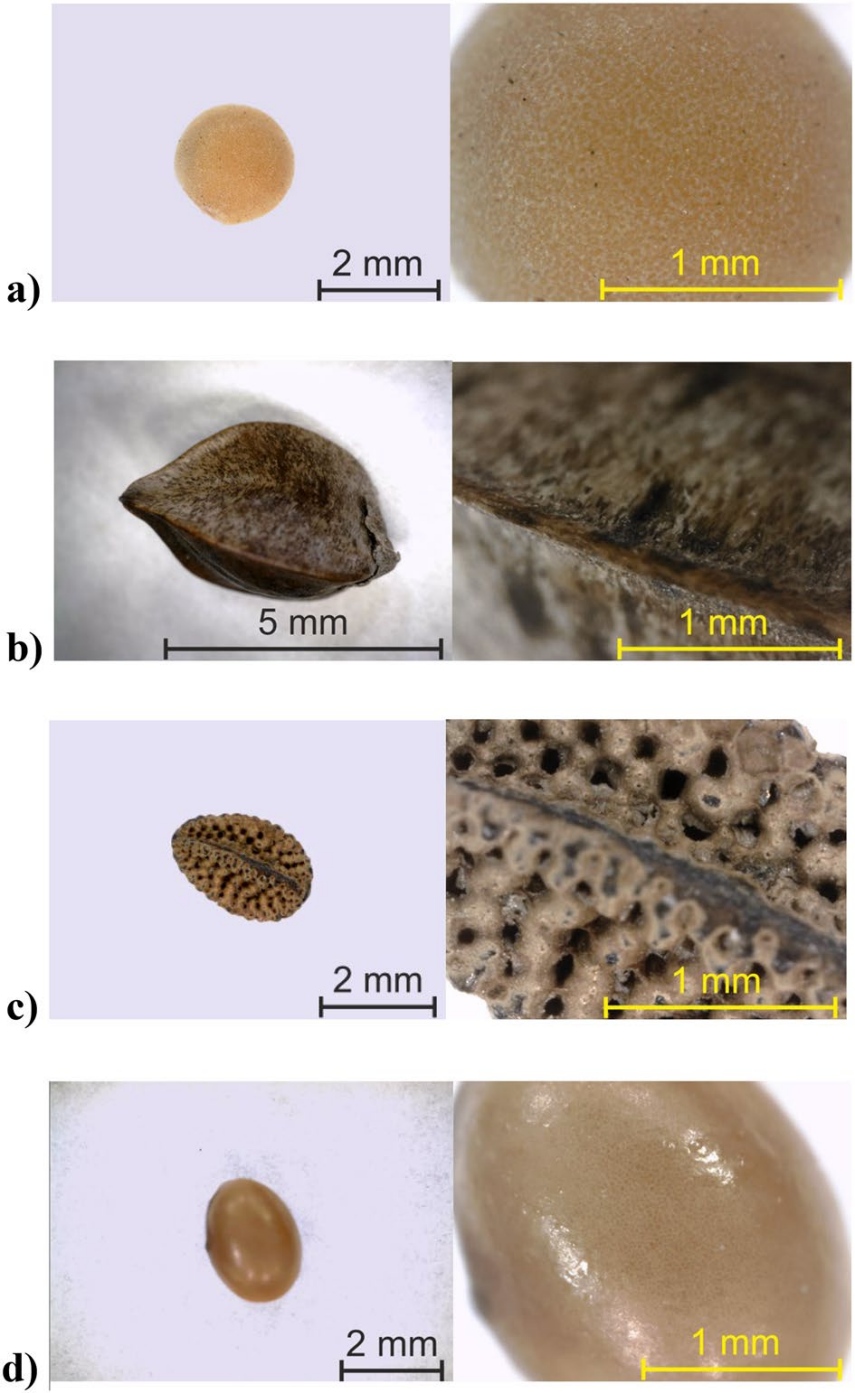
Micrographs of the seeds with magnifications for (**a**) mustard, (**b**) buckwheat, (**c**) phacelia, (**d**) crimson clover respectively.

Figure 1 presents close-up images of the seeds from a digital microscope (Dino-Lite). The quoted in the introduction section external seed characteristics are consistent with those in this study. Seeds sourced from a local certified seed producer before preparing the measuring samples were stored in sealed containers to maintain the integrity of the samples.

For each seed species, five moisture levels were prepared by adding calculated quantities of demineralised water to the respective samples. The first moisture level consisted of seeds of initial water content, where no extra water was added to the samples. In a subsequent step of the sample preparation procedure, the seeds were sealed in an airtight container and kept in a refrigerator at 7 °C ± 1 °C for 5 days. Seeds were stirred/mixed every 6 h to achieve a homogeneous moisture distribution. Before measurements the seeds were removed from the fridge to achieve a temperature of 23 °C ± 1 °C.

The initial moisture content (storage moisture), and other pre-set moisture content levels of seed samples were determined on a fresh weight basis with the oven method (gravimetric method) at 103 °C for 24 h according with the ISTA 2018 reference method for moisture testing. The results were presented in Table 1. The highest moisture values were obtained for phacelia seeds, which are believed to absorb water more readily due to their irregular structure Fig. 1c. The initial moisture content values shown in the table are consistent with the recommended air-free moisture conditions for long-term storage of mustard, crimson clover and buckwheat seeds^50,69^. The study covered the range of seed moisture content that can occur between harvest and storage.

After the seeds water content verification by the oven-drying method the volumetric water content was finally calculated. The volumetric water content (*θ*) for all seed samples was determined by calculating the ratio of the volume of water in each sample to the total wet sample volume placed in a measurement cell and then multiplied by 100 to express it as a percentage. The bulk density of the measured seeds was calculated by weighing the samples and dividing by the volume.

**Table 1:** Mass/gravimetric moisture content values *w* for all seed species for respective moisture level - mean and standard deviation (SD) from three repetitions.

Moisture content level (w)/Seed species	Mustard	Common buckwheat	Tansy phacelia	Crimson clover
I. Initial moisture	7.57 ± 0.09	11.99 ± 0.07	15.03 ± 0.05	8.20 ± 0.01
II. Post-initial moisture	10.87 ± 0.36	15.10 ± 0.20	17.70 ± 0.14	10.57 ± 0.10
III. Middle moisture	14.45 ± 0.29	18.19 ± 0.42	20.89 ± 0.35	14.32 ± 0.67
IV. Pre-maximum moisture	17.36 ± 0.15	21.19 ± 0.16	23.40 ± 0.63	17.35 ± 0.30
V. Maximum moisture	20.17 ± 0.09	22.12 ± 1.22	26.89 ± 0.24	20.49 ± 0.15

### Dielectric spectra measurement in the coaxial cell system

Measurements of dielectric permittivity spectra were carried out in a coaxial-cell system in the frequency range from 20 MHz − 3 GHz. The experiment was performed in laboratory conditions at 23 ± 1 °C. Seed tests were carried out for five different gravimetric moisture content levels (Table 1). Three samples of each seed species with a given moisture content were measured. Measured seeds were placed inside the cell (maximum cell/sample volume 43 cm^3^) between two plastic beads with o-rings.

**Fig. 2:**
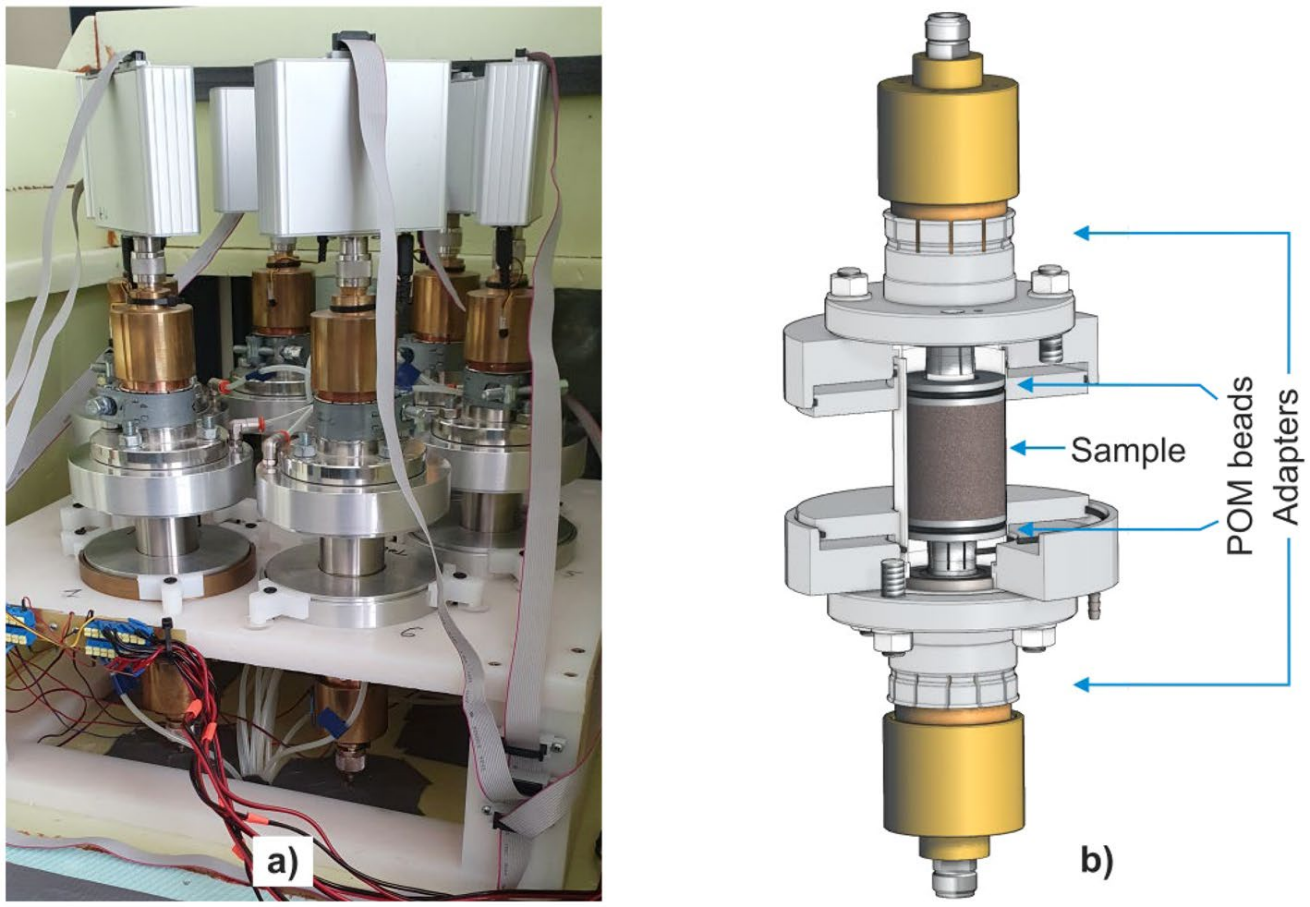
(**a**) Six coaxial cell system, (**b**) cross-section of the coaxial cell.

The coaxial cell system was integrated with a Copper Mountain R60 vector network analyzer (VNA). The coaxial cell system (Fig. 2a,b) is calibrated with an electronic calibration unit (ECU), enabling precise measurement of reflection and transmission parameters, known as S-parameters. Calibration was performed by automatically switching the ECU between various calibration standards such as open, short, offset short, and load in a specified sequence. This procedure enabled recovering the corrected S-parameter matrix from reflection measurements using the mathematical formulas detailed in^70^. The S-parameters of the sample were determined with the use of the transmission matrix formalism as presented in^12^, which enabled subtracting the influence of the air sections of the transmission line and the plastic supports. Then, the complex dielectric permittivity of the samples was extracted with the use of a nonlinear least squares optimization approach implemented in Matlab and presented in detail in^70^. This setup ensured accurate and reliable measurement of the electromagnetic properties of the samples. The more detailed system specifications and calibration procedures, including mathematical formulas, were outlined in^12,70^.

As mentioned in the introduction the loss tangent (tan *δ*) was defined as: tan *δ* = ε″/ε′^21^, and penetration depth (*d*_*p*_) was calculated after obtaining the complex dielectric permittivity of every seed species according with Eq. (1)^30,71^.

1 $$\:{d}_{p}=\frac{c}{2\pi\:f\sqrt{2{\epsilon\:}^{{\prime\:}}\left[\sqrt{1+{\left(\frac{{\epsilon\:}^{{\prime\:}{\prime\:}}}{{\epsilon\:}^{{\prime\:}}}\right)}^{2}}-1\right]}}$$

where *d*_*p*_ = electromagnetic wave penetration depth in m, *f* = frequency in Hz, *c* = speed of light in free space (299 792 458 m s^−1^).

The effective electrical conductivity was defined as $\:\sigma\:={\omega\:\epsilon\:}_{0}\epsilon^{\prime\prime}$, expressed in S/m, where $\:{\epsilon\:}_{0}$ is the permittivity of free space, 8.854 × 10^−12^ F/m, and $\:\omega\:=2\pi\:f$, where *f* is frequency in Hz^21^.

### Seed electrical conductivity test ($\:{EC}_{t}$)

Additionally, the seed electrical conductivity test ($\:{EC}_{t}$) after several months (7 months) storage period in sealed containers in temperature of 7 °C ± 1 °C was carried out for all pre-established moisture levels on samples of 50 seeds for each species soaked in an equal amount of deionised water (75 ml). The soaked samples were kept in closed containers for day at a constant temperature (23 °C ± 1 °C) in a climate chamber and the EC of the soak water was measured after 24 h (ISTA 2018) with multifunction meter CX-701, using a conductivity sensor CDT-2, in µS/cm per gram of initial seed mass.

### Statistical analysis

All measurements were performed in triplicate. The calculation of correlation coefficient (Pearson correlation), linear and polynomial regression on experimental data was performed using R4.2.1 (GNU General Public License). The statistical results were analyzed with one-way analysis of variance (ANOVA) followed by Tukey’s test at the significant level of *p* = 0.05. Assumptions underlying the analytical methods were evaluated with standard diagnostic tools provided by the Rcmdr package in R, specifically Q-Q plots to assess normality and Bartlett’s test to assess homogeneity of variances.

## Results and discussion

### Volumetric water content, bulk density, thousand seed weight and dielectric permittivity results of the studied/tested seeds

The volumetric water content *θ*, bulk density and thousand seed weight results for all seed samples and for respective mass moisture content levels I-V are presented in Table 2. A slightly wider volumetric water content range characterised the crimson clover and mustard seeds. The bulk density *ρ* of the measured samples ranged from 0.73 to 0.89 g/cm^3^, 0.66–0.71 g/cm^3^, 0.57–0.69 g/cm^3^, 0.82–0.92 g/cm^3^ for mustard, buckwheat, phacelia and crimson clover respectively. The thousand seed weight (TSW) of the measured samples ranged from 5.90 to 7.62 g, 27.70–33.50 g, 1.90–2.45 g, 4.08–4.89 g for mustard, buckwheat, phacelia and crimson clover respectively.

**Table 2:** Volumetric water content *θ* corresponding to gravimetric moisture content of the studied seed samples, bulk density and thousand seed weight - mean and SD.

Parameter	Voumetric water content level θ /Seed species	Mustard	Common buckwheat	Tansy phacelia	Crimson clover
Voumetric water content [%]	I. Initial *θ*	5.72 ± 0.19	8.17 ± 0.17	8.79 ± 0.25	6.78 ± 0.13
	II. Post-initial *θ*	8.59 ± 0.32	10.22 ± 0.22	10.42 ± 0.14	8.78 ± 0.13
	III. Middle *θ*	11.95 ± 0.24	12.57 ± 0.41	12.62 ± 0.60	12.13 ± 0.64
	IV. Pre-maximum *θ*	15.01 ± 0.42	14.34 ± 0.19	14.63 ± 0.84	14.89 ± 0.37
	V. Maximum *θ*	16.50 ± 1.17	15.13 ± 0.64	18.34 ± 0.08	18.63 ± 0.13
Bulk density [g/cm^3^]	I. Initial *θ*	0.75 ± 0.02	0.68 ± 0.01	0.58 ± 0.02	0.83 ± 0.01
	II. Post-initial *θ*	0.79 ± 0.01	0.67 ± 0.02	0.59 ± 0.01	0.83 ± 0.01
	III. Middle *θ*	0.82 ± 0.02	0.69 ± 0.02	0.60 ± 0.03	0.84 ± 0.01
	IV. Pre-maximum *θ*	0.86 ± 0.02	0.67 ± 0.01	0.62 ± 0.02	0.86 ± 0.01
	V. Maximum *θ*	0.82 ± 0.06	0.68 ± 0.01	0.68 ± 0.01	0.91 ± 0.01
Thousand seed weight [g]	I. Initial *θ*	6.01 ± 0.10	28.89 ± 1.07	2.00 ± 0.09	4.12 ± 0.03
	II. Post-initial *θ*	6.44 ± 0.45	30.20 ± 1.01	2.05 ± 0.01	4.14 ± 0.08
	III. Middle *θ*	6.48 ± 0.11	31.42 ± 0.25	2.16 ± 0.06	4.43 ± 0.14
	IV. Pre-maximum *θ*	6.79 ± 0.16	32.72 ± 0.76	2.23 ± 0.03	4.57 ± 0.06
	V. Maximum *θ*	7.18 ± 0.42	32.79 ± 0.46	2.33 ± 0.11	4.81 ± 0.10

**Fig. 3:**
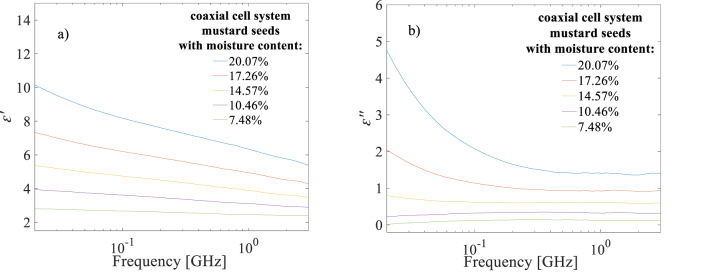
Real (**a**) and imaginary (**b**) part of complex dielectric permittivity spectra, for representative mustard seed species with gravimetric water content (*w*) from 7.48 to 20.07% denoted in the figure respectively.

The real and imaginary part of dielectric permittivity of selected mustard seed species was shown in Fig. 3. As illustrated Fig. 3, the real $\:{\epsilon\:}^{{\prime\:}}$ and imaginary $\:{\epsilon\:}^{{\prime\:}{\prime\:}}$ part of dielectric permittivity of the seed increases with increasing moisture content and decreases with increasing frequency. This trend was observed for all other seed species tested.

### Pearson correlations √ε′, d_p,_ σ, Tan δ and θ features for all seed species

To check potential correlations between features √*ε*′, *tan δ*,* d*_*p*,_ σ and *θ* Pearson’s correlation test for all seed species at specific frequencies 40.68 MHz, 915 MHz and 2.45 GHz useful for radio-frequency and microwave processing^20,30,72^ was performed. Statistically significant (p-value < 0.0001) results were achieved with a strong positive strength for: √*ε*″-*θ*, *tan δ - θ* and σ *- θ* cases considered, while for *d*_*p*_ *- θ* relationship a strong negative correlation was obtained (Table 3).

**Table 3:** Pearson correlations and p-values for *ε*′, *ε*″, √*ε*′, *Tan δ*,* d*_*p*,_ σ and *θ* features for all seed species data at specific frequencies 40.68 MHz, 915 MHz and 2.45 GHz.

	√ε′- θ	tan δ- θ	d_*p*_-θ	σ- θ
40.68 MHz	0.9645	0.8976	-0.7873	0.8621
	< 0.0001	< 0.0001	< 0.0001	< 0.0001
915 MHz	0.9646	0.9148	-0.8089	0.9508
	< 0.0001	< 0.0001	< 0.0001	< 0.0001
2.45 GHz	0.9588	0.9494	-0.8260	0.9567
	< 0.0001	< 0.0001	< 0.0001	< 0.0001

### Seed dielectric permittivity and volumetric water content relationship

**Fig. 4:**
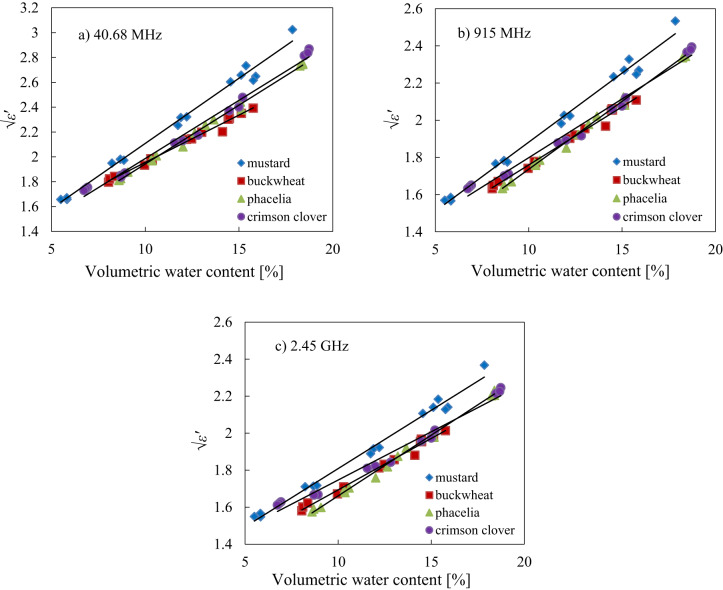
The relationship of the square root of the real part of dielectric permittivity √*ε*′ with volumetric water content *θ* at selected frequencies of 40.68 MHz **(a)**, 915 MHz **(b)** and 2.45 GHz **(c)** for mustard, buckwheat, phacelia and crimson clover seeds, respectively with fitted model of Eq. (2).

As mentioned previously in the introduction section there is a linear relationship between volumetric water content and the square root of the real part of dielectric permittivity, which was also corroborated by the correlation analysis presented above (Table 3). Therefore, a model in the form of Eq. (2) at selected frequencies 40.68 MHz, 915 MHz and 2.45 GHz was fitted to the data of all seed species separately and results were presented on Fig. 4 and in Table 4.

2 $$\:\sqrt{{\epsilon\:}^{{\prime\:}}}\left(\theta\:\right)=A\theta\:+B$$

This study confirmed the strong linear correlation for the analysed parameters of Eq. (2). High coefficient of determination was observed for all analysed seed species, and the best result was found for phacelia seeds, which exhibited the highest coefficient of determination and the lowest root mean square error. The highest permittivity was observed for mustard seeds (Fig. 4).

**Table 4:** Parameters: R2 - coefficient of determination, RMSE - root mean square error and A, B, C parameters of the fitted model of Eqs. (2, 3, 4, 5) for all seeds data at selected frequencies.

Equation		(2) $\:\sqrt{{\epsilon\:}^{{\prime\:}}}\:\left(\theta\:\right)=A\theta\:+B$			(3) $\:{tan}\delta\:\:\left(\theta\:\right)=A\:{\theta\:}^{2}+B\theta\:+C$			(4) $\:{d}_{p}\left(\theta\:\right)=A\:{\theta\:}^{2}+B\theta\:+C$			(5) $\:\sigma\:\left(\theta\:\right)=A{\theta\:}^{2}+B\theta\:+C$		
Species	Parameters/Frequency	40.68 MHz	915 MHz	2.45 GHz	40.68 MHz	915 MHz	2.45 GHz	40.68 MHz	915 MHz	2.45 GHz	40.68 MHz	915 MHz	2.45 GHz
*mustard*	*R* ^2^	0.987	0.986	0.986	0.990	0.982	0.991	0.894	0.932	0.931	0.988	0.975	0.985
	*RMSE*	0.049	0.035	0.031	0.010	0.007	0.006	3.484	0.051	0.024	0.0002	0.003	0.006
	*A*	0.105	0.074	0.062	0.001	-0.0006	-0.0005	0.362	0.006	0.003	5.10e-05	0.0001	0.0004
	*B*	1.058	1.139	1.179	-0.005	0.028	0.029	-10.32	-0.180	-0.086	-0.0006	0.001	0.001
	*C*				0.015	-0.084	-0.101	73.84	1.41	0.654	0.002	-0.005	-0.010
*buckwheat*	*R* ^2^	0.987	0.988	0.986	0.985	0.928	0.98	0.987	0.969	0.991	0.981	0.975	0.981
	*RMSE*	0.023	0.018	0.017	0.004	0.006	0.004	0.368	0.009	0.003	7.382e-05	0.001	0.003
	*A*	0.076	0.062	0.055	0.001	-0.0006	-0.0009	0.008	0.002	0.001	2.941e-05	1.069e-05	2.301e-05
	*B*	1.193	1.134	1.138	-0.032	0.023	0.035	-1.402	-0.075	-0.045	-0.0005	0.002	0.008
	*C*				0.195	-0.040	-0.130	22.88	0.745	0.395	0.002	-0.008	-0.041
*phacelia*	*R* ^2^	0.995	0.996	0.995	0.993	0.904	0.968	0.937	0.962	0.967	0.996	0.985	0.989
	*RMSE*	0.023	0.015	0.015	0.006	0.006	0.007	0.967	0.009	0.005	9.348e-05	0.001	0.004
	*A*	0.094	0.073	0.065	0.0004	-0.0005	-0.0006	0.15	0.001	0.0008	3.139e-05	3.228e-05	0.0002
	*B*	0.994	1.006	1.006	0.011	0.019	0.028	-5.064	-0.049	-0.030	-0.0004	0.0025	0.004
	*C*				-0.077	-0.004	-0.088	44.21	0.581	0.307	0.001	-0.007	-0.021
*crimson clover*	*R* ^2^	0.988	0.982	0.979	0.995	0.993	0.995	0.908	0.965	0.988	0.996	0.997	0.998
	*RMSE*	0.045	0.036	0.032	0.004	0.004	0.004	3.819	0.038	0.009	8.66e-05	0.001	0.002
	*A*	0.094	0.063	0.052	0.0004	-0.0003	3.601e-05	0.386	0.005	0.002	2.629e-05	0.0001	0.0006
	*B*	1.043	1.167	1.226	0.005	0.022	0.014	-12.21	-0.171	-0.070	-0.0003	-2.34e-05	-0.004
	*C*				-0.036	-0.088	-0.056	96.83	1.535	0.637	0.001	-0.001	0.016

### Seed loss tangent characteristics in the 20 MHz-3 GHz frequency range

**Fig. 5:**
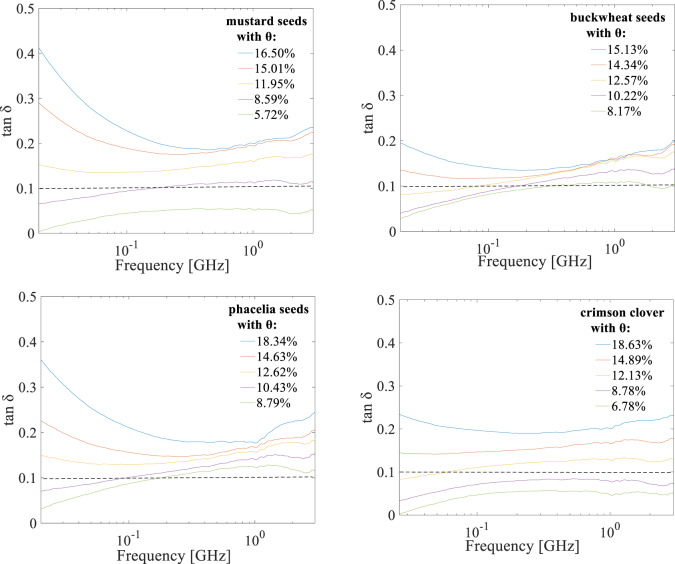
Loss tangent characteristics for mustard, buckwheat, phacelia and crimson clover seeds with different volumetric water content *θ* respectively in the 20 MHz-3 GHz frequency range. The dashed line indicates the division into medium (above the line) microwave absorbing seed samples and low absorbing seed samples (below the line).

The loss tangent determined for all seed species in the analyzed 20 MHz-3 GHz frequency range was shown in Fig. 5.

It occurred that mustard and crimson clover seeds exhibited a wider range of loss tangent values in the analyzed frequency range than the other seeds. The highest loss tangent was noted for mustard.

In general, materials can be classified as high (tan δ > 0.5), medium (0.1–0.5), and low microwave absorbing (< 0.1)^73^. As presented in Fig. 5, the following batches of seeds were classified as medium microwave absorbing material: mustard seeds at volumetric water content *θ* above 11.95%, buckwheat seeds at *θ* above 14.34%, phacelia seeds at *θ* above 12.62% and crimson clover at* θ* above 14.89% respectively, in the whole analyzed frequency range. Mustard and crimson clover seeds with initial water content of 5.72% and 6.78% respectively, and clover seeds with water content of 8.78% were found to be low absorbing samples in the entire frequency range.

However, for seeds at a volumetric water content *θ* of around 8.59% for mustard, at *θ* = 8.17% and 10.22% for buckwheat, at *θ* = 8.79% and 10.43% for phacelia and at *θ* = 12.13% for crimson clover, as the frequency increases, the seeds change from low absorbent to medium absorbent, as observed in Fig. 5. The tested seeds were therefore classified as low and medium microwave absorbing, pointing out that the absorption increases with increasing water content.

### The relationship between loss tangent and volumetric water content

**Fig. 6:**
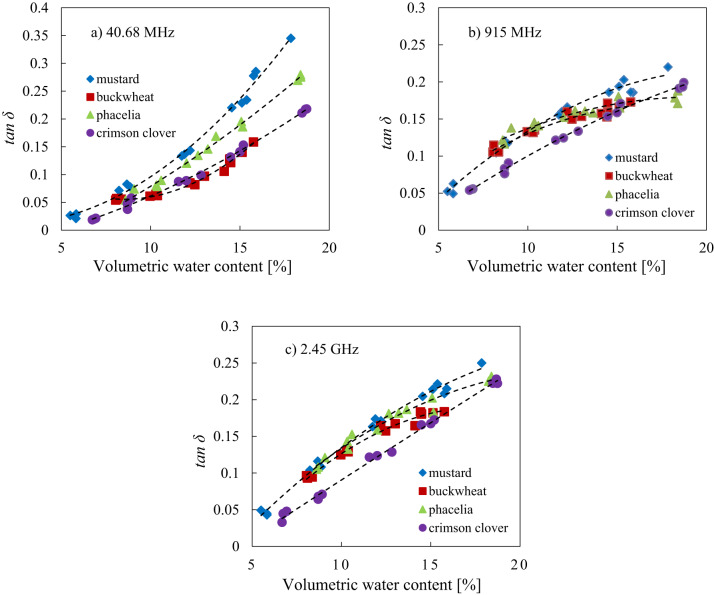
The relationship between the loss tangent $\:({tan}\delta\:)$ and volumetric water content (*θ*) at selected frequencies of 40.68 MHz **(a)**, 915 MHz **(b)** and 2.45 GHz **(c)** for mustard, buckwheat, phacelia and crimson clover seeds respectively, with fitted model of Eq. (3).

To test the ability of the various moisture seed material to convert microwaves energy into heat at frequencies specific for heating purposes (40.68 MHz, 915 MHz and 2.45 GHz), a model in the form of Eq. (3) was fitted to the data and results were presented in Fig. 6 and Table 4.

3 $$\:{tan}\delta\:\:\left(\theta\:\right)=A\:{\theta\:}^{2}+B\theta\:+C\:$$

A good fit of Eq. (3) for analyzed parameters was found for mustard, buckwheat, phacelia and crimson clover seeds (R^2^ above 0.9 - Table 4; Fig. 6a–c) for all analyzed frequencies. It was found that the loss tangent increased with increasing volumetric water content (Fig. 6a–c). Moreover, similar slopes of the curves from fitting for phacelia and buckwheat seeds (Fig. 6b, c), as well as for mustard and crimson clover seeds were observed (Fig. 6b).

### Penetration depth in the 20 MHz-3 GHz frequency range

**Fig. 7:**
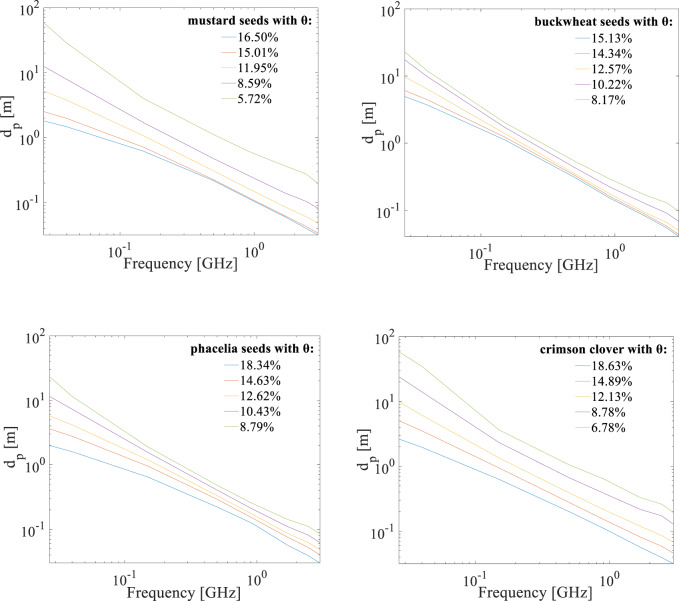
Electric field penetration depth values for mustard, buckwheat, phacelia and crimson clover seeds with different volumetric water content *θ* respectively in the 20 MHz-3 GHz frequency range.

The electric field penetration depth for all seed species in the analyzed 20 MHz-3 GHz frequency range was shown in Fig. 7. The penetration depth decreases with increasing frequency. The highest penetration depth characterises mustard and crimson clover seeds.

### Electric field penetration depth and volumetric water content relationship

**Fig. 8:**
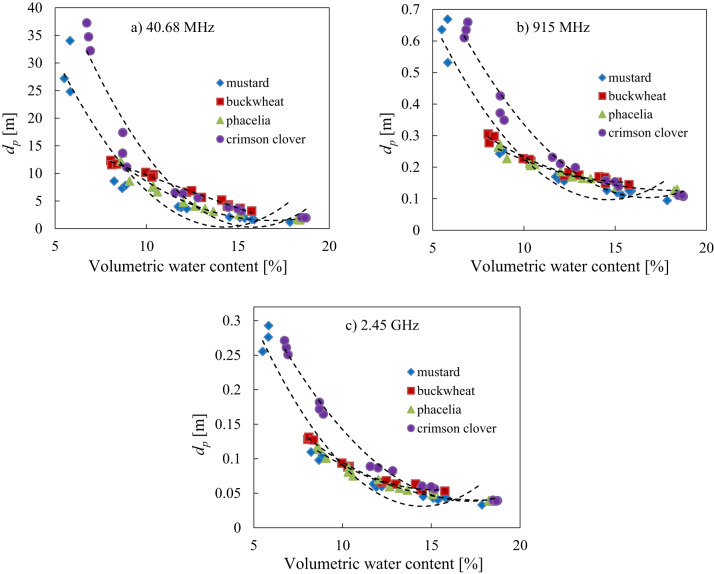
The relationship between the penetration depth $\:{d}_{p}$ and volumetric water content *θ* at selected frequencies of 40.68 MHz **(a)**, 915 MHz **(b)** and 2.45 GHz **(c)** for mustard, buckwheat, phacelia and crimson clover seeds, respectively with fitted model of Eq. (4).

In order to explore the relationship between seed moisture and penetration depth of the various moisture seed material at a specific frequencies using for heating purposes (40.68 MHz, 915 MHz and 2.45 GHz), a model in the form of Eq. (4) was fitted to the data and the results were presented on Fig. 8 and in Table 4.

4 $$\:{d}_{p}\left(\theta\:\right)=A{\theta\:}^{2}+B\theta\:+C$$

This study confirmed the strong relationship for the analysed parameters of Eq. (4) for frequencies of 40.68 MHz, 915 MHz and 2.45 GHz, for all seed species with the best result found for buckwheat seeds in 2.45 GHz with the highest coefficient of determination and the lowest root mean square error (Table 4). As illustrated in Fig. 8a–c seeds with higher water content had lower penetration depth. The highest penetration depth for mustard and crimson clover seeds at initial moisture content was observed (Fig. 8a–c), which is probably related to the lower volumetric water content of these seed species compared to the other two. With the 40.68 MHz frequency analyzed, the highest penetration depth parameter was found as compared to the results for frequency of 915 MHz and 2.45 GHz for all seed species tested across the entire moisture range. As known from previous studies the penetration depth for pure water at room temperature is 1.04 cm at 2.45 GHz^74^. Values of the same order (~ 4 cm) were observed for the crimson clover, mustard and phacelia seeds with the highest water content.

Determining the electric field penetration depth can be useful in selecting/adjusting parameters that can optimise the uniformity and efficiency of a potential application of microwave heating or disinfestation or mould control in respective seed species.

In conclusion, at the lowest examined frequency (40.68 MHz), all seed species have higher ability to convert the electromagnetic energy to heat with better penetration, and when the frequency increases, energy is absorbed by several centimetres of seed layer (for 915 MHz) and up to a maximum of a few centimetres of seed layer (for 2.45 GHz), resulting in lower penetration.

### Effective electrical conductivity of the seeds in the 20 MHz-3 GHz frequency range

**Fig. 9:**
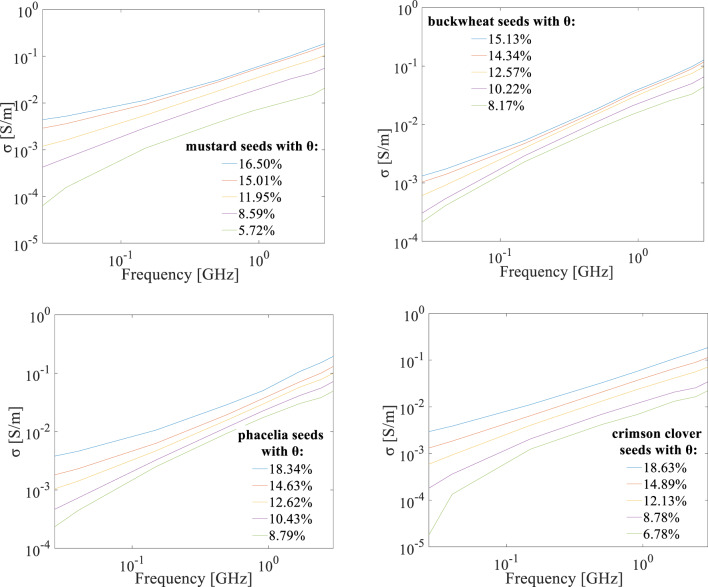
Effective electrical conductivity as a function of frequency for mustard, buckwheat, phacelia and crimson clover seeds with different volumetric water content *θ* respectively in the 20 MHz-3 GHz frequency range.

Electrical conductivity measures a material’s ability to conduct electricity, which is influenced by the presence of ions and water. An increase in effective electrical conductivity with increasing volumetric water content and frequency was found for all seed species in the analysed frequency range. The smallest range of conductivity changes over the entire analyzed frequency range was observed for buckwheat seeds (Fig. 9).

### Relationship between seed effective electrical conductivity and water content

**Fig. 10:**
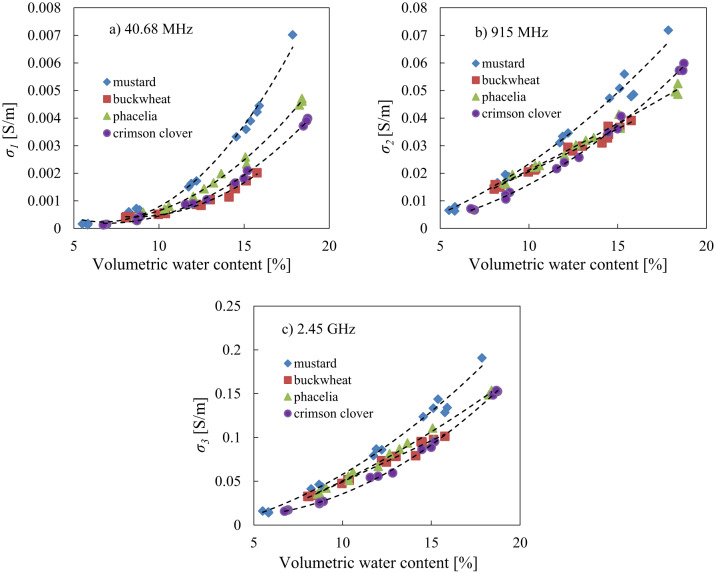
The relationship between the effective electrical conductivity $\:\sigma\:$ and volumetric water content *θ* at selected frequencies of 40.68 MHz **(a)**, 915 MHz **(b)** and 2.45 GHz **(c)** for mustard, buckwheat, phacelia and crimson clover seeds respectively, with fitted model of Eq. (5).

The relationship of the effective electrical conductivity $\:\sigma\:$ vs. volumetric water content *θ* - Eq. (5) at selected/specific frequencies of 40.68 MHz, 915 MHz and 2.45 GHz and parameters of the fitted model of Eq. (5) for mustard, buckwheat, phacelia and crimson clover seeds shown in Fig. 10; Table 4.

$$\sigma\:\left(\theta\:\right)=A{\theta\:}^{2}+B\theta\:+C$$

As the volumetric water content of the seeds increases, the effective electrical conductivity also increases. The highest effective electrical conductivity values were found for the seeds of crimson clover, phacelia and mustard for the highest water content level at 2.45 GHz (Fig. 10). Based on the model fitting (Eq. 5) results high coefficients of determination were found for all analyzed seed species at frequencies of 40.68 MHz, 915 MHz and 2.45 GHz, with the best results for crimson clover seeds (see Table 4).

### The electrical conductivity test $\:{(EC}_{t})\:$results

**Fig. 11:**
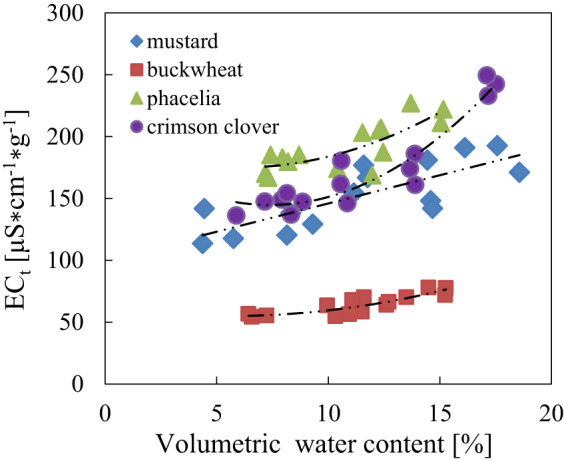
The relationship of the electrical conductivity $\:{\mathrm{E}\mathrm{C}}_{\mathrm{t}}$ and volumetric water content after 7 months of storage for mustard, buckwheat, phacelia and crimson clover seeds respectively, with fitted trend line.

The results of conductivity test $\:{(EC}_{t})\:$performed in accordance with the guidelines [ISTA 2018] as a function of volumetric water content are shown in Fig. 11. An increase in conductivity with increasing water content after 7 months storage period for phacelia, crimson clover, mustard and buckwheat seeds, respectively was observed. As can be seen in the Fig. 11, phacelia, clover and mustard seeds had higher conductivity compared to buckwheat seeds and thus seeds of these three species had lower vigour after storage period unlike buckwheat seeds, which were characterised by a slight increase in conductivity with an increase in volumetric water content and thus better seed condition.

Statistically significant positive correlations (strong relationship) were found for all analazyed $\:{\mathrm{E}\mathrm{C}}_{\mathrm{t}}$ – *θ*, $\:{\mathrm{E}\mathrm{C}}_{\mathrm{t}}$– σ_1_, $\:{\mathrm{E}\mathrm{C}}_{\mathrm{t}}$ – σ_2_, $\:{\mathrm{E}\mathrm{C}}_{\mathrm{t}}$ – σ_3_ pairs among the seed species studied, where $\:{\mathrm{E}\mathrm{C}}_{\mathrm{t}}$ *–* data from the conductivity test performed and σ_1_, σ_2_, σ_3_ were calculated from data for respective seed species at specific frequencies 40.68 MHz (σ_1_), 915 MHz (σ_2_), and 2.45 GHz (σ_3_) (Table 5). The highest correlation was observed for crimson clover seeds and the lowest for mustard for conductivity from the test and that calculated at 40 MHz ($\:{\mathrm{E}\mathrm{C}}_{\mathrm{t}}$ – σ_1_ relationship in Table 5). Once a correlation has been established for the electrical conductivity measured and calculated seed conductivity at specific frequencies, the other one can also be used to assess seed quality, particularly for crimson clover and buckwheat seed species where the strongest correlations were found.

**Table 5:** Pearson correlations and p-values for $\:{\mathrm{E}\mathrm{C}}_{\mathrm{t}}$ – *θ* ,$\:\:{\mathrm{E}\mathrm{C}}_{\mathrm{t}}$ – σ_1_, $\:{\mathrm{E}\mathrm{C}}_{\mathrm{t}}$ – σ_2_, $\:{\mathrm{E}\mathrm{C}}_{\mathrm{t}}$ – σ_3_ pairs where $\:{\mathrm{E}\mathrm{C}}_{\mathrm{t}}$ *–* data from the conductivity test performed and σ_1_, σ_2_, σ_3_ were calculated from data for respective seed species at specific frequencies 40.68 MHz (σ_1_), 915 MHz (σ_2_), and 2.45 GHz (σ_3_) for the tested moisture content ranges.

	$\:{\mathrm{E}\mathrm{C}}_{\mathrm{t}}$ – θ	$\:{\mathrm{E}\mathrm{C}}_{\mathrm{t}}$ – σ_1_	$\:{\mathrm{E}\mathrm{C}}_{\mathrm{t}}$ – σ_2_	$\:{\mathrm{E}\mathrm{C}}_{\mathrm{t}}$ – σ_3_
*mustard*	0.7936 0.0004	0.6085 0.0161	0.7531 0.0012	0.7444 0.0015
*buckwheat*	0.8378 < 0.0001	0.8700 < 0.0001	0.8621 < 0.0001	0.8634 < 0.0001
*phacelia*	0.7782 < 0.0001	0.8470 < 0.0001	0.8289 0. 0001	0.8428 < 0.0001
*crimson clover*	0.8646 < 0.0001	0.9268 < 0.0001	0.8897 < 0.0001	0.9012 < 0.0001

### Comparison of the $\: \sqrt {\varepsilon ^{\prime}}, tan\delta$, d_p_, σ parameters of all seed species for three specific frequencies (40.68 MHz, 915 MHz, 2.45 GHz) at ~15% volumetric water content

To determine whether different seed species exhibit differences in dielectric properties at a fixed volumetric water content (~ 15%), an analysis of variance (ANOVA) was conducted on the following parameters: the square root of the real part of dielectric permittivity (√ε′), loss tangent (tanδ), penetration depth (d_p_), and effective electrical conductivity (σ). The analyses were performed at the frequencies 40.68 MHz, 915 MHz, and 2.45 GHz.

**Table 6:** One-way analysis of variances (ANOVA) of the $\: \sqrt {\varepsilon ^{\prime}}, tan\delta$, *d*_*p*_, *σ* parameters performed at three specific frequencies (40.68 MHz, 915 MHz, 2.45 GHz) at ~15% volumetric water content seed species.

Species	Parameter	Frequency			p-value
		40.68 MHz	915 MHz	2.45 GHz	
*Mustard*	√*ε*′	2.67 ± 0.07 C a	2.28 ± 0.05 B b	2.14 ± 0.04 A c	< 0.0001
	$\:{tan}\delta\:$	0.23 ± 0.01 B c	0.19 ± 0.01 A b	0.21 ± 0.01 AB c	0.006
	*d*_*p*_	1.98 ± 0.11 B a	0.12 ± 0.01 A a	0.043 ± 0.002 A a	< 0.0001
	*σ*	0.003 ± 0.0002 A b	0.051 ± 0.004 B b	0.133 ± 0.010 C b	< 0.0001
*Buckwheat*	√*ε*′	2.35 ± 0.04 C a	2.08 ± 0.03 B a	1.98 ± 0.001 A ab	< 0.0001
	$\:{tan}\delta\:$	0.14 ± 0.02 A a	0.17 ± 0.01 ABa	0.18 ± 0.001 B ab	0.011
	*d*_*p*_	3.69 ± 0.56 B b	0.15 ± 0.01 A b	0.054 ± 0.001 A b	< 0.0001
	*σ*	0.001 ± 0.0002 A a	0.036 ± 0.002 B a	0.097 ± 0.003 C a	< 0.0001
*Phacelia*	√*ε*′	2.37 ± 0.07 B a	2.08 ± 0.05 A a	1.97 ± 0.04 A b	0.0003
	$\:{tan}\delta\:$	0.18 ± 0.01 A b	0.17 ± 0.01 A a	0.19 ± 0.01 A b	0.128
	*d*_*p*_	2.77 ± 0.29 B ab	0.15 ± 0.01 A b	0.052 ± 0.004 A b	< 0.0001
	*σ*	0.002 ± 0.0003 A a	0.036 ± 0.004 B a	0.100 ± 0.008 C a	< 0.0001
*Crimson clover*	√*ε*′	2.42 ± 0.06 B a	2.08 ± 0.03 A a	1.98 ± 0.03 A a	< 0.0001
	$\:{tan}\delta\:$	0.14 ± 0.01 A a	0.17 ± 0.01 B a	0.17 ± 0.003 B a	0.011
	*d*_*p*_	3.52 ± 0.34 B b	0.15 ± 0.01 A b	0.059 ± 0.002 A b	< 0.0001
	*σ*	0.001 ± 0.0002 A a	0.037 ± 0.003 B a	0.090 ± 0.004 C a	< 0.0001
p-value		0.0007	0.0007	0.0009	
p-value		< 0.0001	0.0129	0.0003	
p-value		0.0015	0.009	0.0003	
p-value		0.00012	0.0026	0.0004	

Explanations: p-value – significance level, means with the same letter mean lack of significance difference at α < 0.05; uppercase letters indicate comparison of respective parameters: √*ε*′, $\:{tan}\delta\:$, *d*_*p*_, *σ* at three specific frequencies for each seed species separately; lowercase letters indicate comparison of respective parameters:√*ε*′, $\:{tan}\delta\:$, *d*_*p*_, *σ* for all seed species at a given frequency.

Considering √*ε*′ quantity, statistically significant higher values were observed at lower frequencies (40.68 MHz and 915 MHz) for mustard and buckwheat. For phacelia and crimson clover seeds, the highest values were noted at 40.68 MHz. Comparing √*ε*′ between seed species, no significant differences were found at 40.68 MHz, while at higher frequencies significant higher values were observed for mustard seeds. Significantly the highest $\:{tan}\delta\:$ was recorded for mustard seeds at 40.68 MHz. $\:{Tan}\delta\:$ values varied by frequency for all seeds tested except for phacelia, significantly lower $\:{tan}\delta\:$ was observed at 40.68 MHz for buckwheat and crimson clover. Comparing the $\:{tan}\delta\:$ between all seed species at respective frequencies, significantly higher $\:{\:tan}\delta\:$ was recorded for mustard. Penetration depth for individual seed species was highest at 40.68 MHz and significantly decreased with increasing frequency. Penetration depth values were found to be significantly lower for mustard seeds among all analysed seed species at the respective frequencies. Conductivity increased statistically significantly with increasing frequency. Statistically higher conductivity in relation to the other seed species was found for mustard seeds at all analysed frequencies with the highest value at 2.45 GHz (Table 6).

As a result, both frequency and seed species were found to influence the analysed parameters (Table 6). Moreover, it was found that for a given seed water content of 15% for all seed species analysed, the examined √*ε*′, $\:{tan}\delta\:$, *d*_*p*_, *σ* parameters differed, and therefore the respective seeds were found to have their own specific dielectric characteristics. Mustard seeds with a given moisture content of ~ 15% compared to the other analysed seed species analysed turned out to possess the highest absorbing properties with the lowest penetration depth at the same time. Therefore, these seeds will require the selection of slightly different parameters at potential processing/heating with radio-frequency or microwave.

The complex dielectric permittivity measured in this study, as well as other dielectric parameters derived from it, pertain to the bulk seed samples, i.e. the medium consisting of the actual seeds and air in the spaces between the seeds. One may determine the dielectric properties of the seed matter using various dielectric mixing models, e.g. those presented in^11^. Here, the focus was placed on bulk quantities, because of the practical importance, as microwave treatments or moisture content measurements are conducted on batches of seeds that naturally include air. Bulk density influences these dielectric parameters, and in general should be taken into account. However, there are also density-independent microwave moisture measurement techniques that utilize the real and imaginary parts of bulk dielectric permittivity of seeds, e.g. the approach described in^75^. The complex dielectric permittivity of the seeds obtained with the use of the coaxial cell system may be used in this method.

## Summary

In conclusion the dielectric parameters √*ε*′, $\:{tan}\delta\:$, *d*_*p*_, *σ* of the analysed seeds of mustard, buckwheat, phacelia and crimson clover are dependent on factors such as water content and frequency. Moreover, dielectric properties vary considerably among different kinds of seed and species. The square root of the real part of complex dielectric permittivity as well as effective electrical conductivity of seeds as a parameter correlating with water content can be useful in assessing seeds’ quality and monitoring changes during storage under different conditions. The paper shows that the dielectric loss tangent and the penetration depth of the seed vary by species. The results of the present study may be useful in determining the optimal conditions for seeds processing with the use of electromagnetic waves.

## Data Availability

The datasets generated during and/or analysed during the current study are available from the corresponding author on reasonable request.
